# Supplementary material for: The Protective Role of 28-Homobrassinolide and Glomus versiforme in Cucumber to Withstand Saline Stress
Source: Plants (Basel). 2019 Dec 26;9(1):42. doi: 10.3390/plants9010042 (PMC7020224; doi:10.3390/plants9010042)
Supplement: Supplementary file 1 [file plants-09-00042-s001.pdf]

Supplemntary materials (ANOVA)

S1a: Analysis of Variance Table for plant length (Cv Jinyou #1)

Source	DF	SS	MS	F	P
r	2	25.69	12.85		
salt	1	4320.17	4320.17	278.47	0.0036
Error r*salt	2	31.03	15.51		
Treat	3	254.07	84.69	4.78	0.0204
salt*Treat	3	25.09	8.36	0.47	0.1074
Error r*salt*Treat	12	212.61	17.72		
Total	23	4868.67			

Grand Mean 84.910
CV(r*salt) 4.64
CV(r*salt*Treat) 4.96

S2a: Analysis of Variance Table for Root length (Cv Jinyou #1)

Source	DF	SS	MS	F	P
r	2	42.03	21.015		
salt	1	850.37	850.374	4085.15	0.0002
Error r*salt	2	0.42	0.208		
Treat	3	115.27	38.422	3.56	0.0474
salt*Treat	3	87.90	29.300	2.72	0.0713
Error r*salt*Treat	12	129.41	10.784		
Total	23	1225.40			

Grand Mean 47.870
CV(r*salt) 0.95
CV(r*salt*Treat) 6.86

S3a: Analysis of Variance Table for Shoot fresh weight (Cv Jinyou #1)

Source	DF	SS	MS	F	P
r	2	83.7	41.8		
salt	1	12795.6	12795.6	331.17	0.0030
Error r*salt	2	77.3	38.6		
Treat	3	1970.9	657.0	11.40	0.0008
salt*Treat	3	880.7	293.6	5.09	0.0168
Error r*salt*Treat	12	691.8	57.7		
Total	23	16499.9			

Grand Mean 96.133
CV(r*salt) 6.47
CV(r*salt*Treat) 7.90

S4a: Analysis of Variance Table for Shoot dry weight (Cv Jinyou #1)

Source	DF	SS	MS	F	P
r	2	39.394	19.697		
salt	1	266.267	266.267	169.38	0.0059
Error r*salt	2	3.144	1.572		
Treat	3	51.337	17.112	3.68	0.0436
salt*Treat	3	4.067	1.356	0.29	0.8309
Error r*salt*Treat	12	55.851	4.654		
Total	23	420.059			

Grand Mean 13.873

CV(r*salt) 9.04
CV(r*salt*Treat) 15.55

S5a: Analysis of Variance Table for Root fresh weight (Cv Jinyou #1)

Source	DF	SS	MS	F	P
r	2	1.830	0.915		
salt	1	405.000	405.000	48.42	0.0200
Error r*salt	2	16.728	8.364		
Treat	3	62.290	20.763	4.50	0.0245
salt*Treat	3	15.139	5.046	1.09	0.3891
Error r*salt*Treat	12	55.334	4.611		
Total	23	556.321			

Grand Mean 15.617
CV(r*salt) 18.52
CV(r*salt*Treat) 13.75

S6a: Analysis of Variance Table for Root dry weight (Cv Jinyou #1)

Source	DF	SS	MS	F	P
r	2	0.2379	0.1190		
salt	1	25.6473	25.6473	100.59	0.0098
Error r*salt	2	0.5099	0.2550		
Treat	3	2.5374	0.8458	4.22	0.0297
salt*Treat	3	0.2144	0.0715	0.36	0.7854
Error r*salt*Treat	12	2.4062	0.2005		
Total	23	31.5533			

Grand Mean 2.3088
CV(r*salt) 21.87
CV(r*salt*Treat) 19.40

S7a: Analysis of Variance Table for Chl a (Cv Jinyou #1)

Source	DF	SS	MS	F	P
r	2	0.1078	0.0539		
salt	1	32.4242	32.4242	164.66	0.0060
Error r*salt	2	0.3938	0.1969		
Treat	3	15.3368	5.1123	32.09	0.0000
salt*Treat	3	7.1457	2.3819	14.95	0.0002
Error r*salt*Treat	12	1.9119	0.1593		
Total	23	57.3203			

Grand Mean 15.810
CV(r*salt) 2.81
CV(r*salt*Treat) 2.52

S8a: Analysis of Variance Table for chl b (Cv Jinyou #1)

Source	DF	SS	MS	F	P
r	2	0.0390	0.0195		
salt	1	10.4830	10.4830	2214.83	0.0005
Error r*salt	2	0.0095	0.0047		
Treat	3	1.8355	0.6118	20.51	0.0001
salt*Treat	3	0.8517	0.2839	9.52	0.0017
Error r*salt*Treat	12	0.3580	0.0298		
Total	23	13.5768			

Grand Mean 3.7937

CV(r*salt) 1.81
CV(r*salt*Treat) 4.55

S9a: Analysis of Variance Table for total chl (Cv Jinyou #1)

Source	DF	SS	MS	F	P
r	2	0.073	0.0364		
salt	1	79.780	79.7801	325.51	0.0031
Error r*salt	2	0.490	0.2451		
Treat	3	27.663	9.2209	63.88	0.0000
salt*Treat	3	12.369	4.1231	28.56	0.0000
Error r*salt*Treat	12	1.732	0.1443		
Total	23	122.107			

Grand Mean 19.603
CV(r*salt) 2.53
CV(r*salt*Treat) 1.94

S10a: Analysis of Variance Table for Root activity (Cv Jinyou #1)

Source	DF	SS	MS	F	P
r	2	0.012	0.006		
salt	1	508.355	508.355	456.80	0.0022
Error r*salt	2	2.226	1.113		
Treat	3	29.895	9.965	14.75	0.0002
salt*Treat	3	6.892	2.297	3.40	0.0535
Error r*salt*Treat	12	8.108	0.676		
Total	23	555.487			

Grand Mean 17.948
CV(r*salt) 5.88
CV(r*salt*Treat) 4.58

S11a: Analysis of Variance Table for Electrolyte leakage after 20 days (Cv Jinyou #1)

Source	DF	SS	MS	F	P
r	2	3.02	1.51		
salt	1	1130.98	1130.98	1734.05	0.0006
Error r*salt	2	1.30	0.65		
Treat	3	65.70	21.90	11.65	0.0007
salt*Treat	3	129.57	43.19	22.98	0.0000
Error r*salt*Treat	12	22.56	1.88		
Total	23	1353.13			

Grand Mean 20.459
CV(r*salt) 3.95
CV(r*salt*Treat) 6.70

S12a: Analysis of Variance Table for Electrolyte leakage after 40 days (Cv Jinyou #1)

Source	DF	SS	MS	F	P
r	2	2.07	1.036		
salt	1	865.84	865.836	270.12	0.0037
Error r*salt	2	6.41	3.205		
Treat	3	127.95	42.649	67.66	0.0000
salt*Treat	3	69.16	23.052	36.57	0.0000
Error r*salt*Treat	12	7.56	0.630		
Total	23	1078.99			

Grand Mean 13.287
CV(r*salt) 13.47
CV(r*salt*Treat) 5.98

S13a: Analysis of Variance Table for Leaf relative water content after 20 days (Cv Jinyou #1)

Source	DF	SS	MS	F	P
r	2	36.25	18.124		
salt	1	950.44	950.444	1467.93	0.0007
Error r*salt	2	1.29	0.647		
Treat	3	542.01	180.669	36.34	0.0000
salt*Treat	3	72.98	24.328	4.89	0.0190
Error r*salt*Treat	12	59.66	4.972		
Total	23	1662.64			

Grand Mean 73.229
CV(r*salt) 1.10
CV(r*salt*Treat) 3.04

S14a: Analysis of Variance Table for Leaf relative water content after 40 days (Cv Jinyou #1)

Source	DF	SS	MS	F	P
r	2	36.57	18.29		
salt	1	2861.30	2861.30	267.21	0.0037
Error r*salt	2	21.42	10.71		
Treat	3	131.51	43.84	16.83	0.0001
salt*Treat	3	32.03	10.68	4.10	0.0322
Error r*salt*Treat	12	31.25	2.60		
Total	23	3114.08			

Grand Mean 49.126
CV(r*salt) 6.66
CV(r*salt*Treat) 3.28

S15a: Analysis of Variance Table for photosynthesis (Cv Jinyou #1)

Source	DF	SS	MS	F	P
r	2	1.1078	0.5539		
salt	1	20.5350	20.5350	5457.81	0.0002
Error r*salt	2	0.0075	0.0038		
Treat	3	1.5649	0.5216	1.67	0.2252
salt*Treat	3	0.6769	0.2256	0.72	0.5568
Error r*salt*Treat	12	3.7388	0.3116		
Total	23	27.6309			

Grand Mean 5.3267
CV(r*salt) 1.15
CV(r*salt*Treat) 10.48

S16a: Analysis of Variance Table for stomatal conductance (Cv Jinyou #1)

Source	DF	SS	MS	F	P
r	2	0.00028	0.00014		
salt	1	0.10868	0.10868	1187.18	0.0008
Error r*salt	2	0.00018	0.00009		
Treat	3	0.00648	0.00216	2.31	0.1281

salt*Treat	3	0.00086	0.00029	0.31	0.8207
Error r*salt*Treat	12	0.01123	0.00094		
Total	23	0.12771			

Grand Mean 0.1918
CV(r*salt) 4.99
CV(r*salt*Treat) 15.95

S17a: Analysis of Variance Table for intercellular Carbon dioxide conc. (Cv Jinyou #1)

Source	DF	SS	MS	F	P
r	2	27.77	13.89		
salt	1	4743.28	4743.28	2461.91	0.0004
Error r*salt	2	3.85	1.93		
Treat	3	544.12	181.37	19.60	0.0001
salt*Treat	3	79.88	26.63	2.88	0.0802
Error r*salt*Treat	12	111.06	9.26		
Total	23	5509.98			

Grand Mean 379.06
CV(r*salt) 0.37
CV(r*salt*Treat) 0.80

S18a: Analysis of Variance Table for transpiration rate (Cv Jinyou #1)

Source	DF	SS	MS	F	P
r	2	0.0181	0.0090		
salt	1	12.0706	12.0706	31797.6	0.0000
Error r*salt	2	0.0008	0.0004		
Treat	3	0.6291	0.2097	42.19	0.0000
salt*Treat	3	0.2374	0.0791	15.92	0.0002
Error r*salt*Treat	12	0.0596	0.0050		
Total	23	13.0155			

Grand Mean 1.5014
CV(r*salt) 1.30
CV(r*salt*Treat) 4.70

S19a: Analysis of Variance Table for Superoxide dismutase (SOD) after 10 days (Cv Jinyou #1)

Source	DF	SS	MS	F	P
r	2	4676.6	2338.3		
salt	1	62899.8	62899.8	103.56	0.0095
Error r*salt	2	1214.8	607.4		
Treat	3	10356.3	3452.1	5.11	0.0165
salt*Treat	3	8379.8	2793.3	4.14	0.0314
Error r*salt*Treat	12	8100.6	675.1		
Total	23	95627.9			

Grand Mean 378.98
CV(r*salt) 6.50
CV(r*salt*Treat) 6.86

S20a: Analysis of Variance Table for Superoxide dismutase (SOD) after 20 days (Cv Jinyou #1)

Source	DF	SS	MS	F	P
r	2	2889	1445		

salt	1	321878	321878	2174.11	0.0005
Error r*salt	2	296	148		
Treat	3	11790	3930	3.22	0.0613
salt*Treat	3	8279	2760	2.26	0.1336
Error r*salt*Treat	12	14643	1220		
Total	23	359775			

Grand Mean 464.05
CV(r*salt) 2.62
CV(r*salt*Treat) 7.53

S21a: Analysis of Variance Table for Superoxide dismutase (SOD) after 30 days (Cv Jinyou #1)

**Source**	**DF**	**SS**	**MS**	**F**	**P**
r	2	3264	1632		
salt	1	339116	339116	280.39	0.0035
Error r*salt	2	2419	1209		
Treat	3	5829	1943	1.77	0.2067
salt*Treat	3	3707	1236	1.12	0.3780
Error r*salt*Treat	12	13189	1099		
Total	23	367524			

Grand Mean 464.96
CV(r*salt) 7.48
CV(r*salt*Treat) 7.13

S22a: Analysis of Variance Table for Superoxide dismutase (SOD) after 40 days (Cv Jinyou #1)

**Source**	**DF**	**SS**	**MS**	**F**	**P**
r	2	25	13		
salt	1	284511	284511	136.54	0.0072
Error r*salt	2	4168	2084		
Treat	3	7461	2487	2.08	0.1560
salt*Treat	3	7054	2351	1.97	0.1725
Error r*salt*Treat	12	14328	1194		
Total	23	317546			

Grand Mean 458.74
CV(r*salt) 9.95
CV(r*salt*Treat) 7.53

S23a: Analysis of Variance Table for Peroxidase (POD) after 10 days (Cv Jinyou #1)

**Source**	**DF**	**SS**	**MS**	**F**	**P**
r	2	538	269		
salt	1	1072627	1072627	2991.85	0.0003
Error r*salt	2	717	359		
Treat	3	27457	9152	5.63	0.0121
salt*Treat	3	8912	2971	1.83	0.1961
Error r*salt*Treat	12	19522	1627		
Total	23	1129774			

Grand Mean 945.62
CV(r*salt) 2.00
CV(r*salt*Treat) 4.27

S24a: **Analysis of Variance Table for Peroxidase (POD) after 20 days (Cv Jinyou #1)**

Source	DF	SS	MS	F	P
r	2	3962	1981		
salt	1	1354302	1354302	1808.09	0.0006
Error r*salt	2	1498	749		
Treat	3	19999	6666	6.32	0.0081
salt*Treat	3	14842	4947	4.69	0.0217
Error r*salt*Treat	12	12662	1055		
Total	23	1407264			

Grand Mean 996.02
CV(r*salt) 2.75
CV(r*salt*Treat) 3.26

S25a: **Analysis of Variance Table for Peroxidase (POD) after 30 days (Cv Jinyou #1)**

Source	DF	SS	MS	F	P
r	2	1642	821		
salt	1	1212157	1212157	3033.95	0.0003
Error r*salt	2	799	400		
Treat	3	9950	3317	2.07	0.1571
salt*Treat	3	7642	2547	1.59	0.2426
Error r*salt*Treat	12	19184	1599		
Total	23	1251375			

Grand Mean 991.21
CV(r*salt) 2.02
CV(r*salt*Treat) 4.03

S26a: **Analysis of Variance Table for Peroxidase (POD) after 40 days (Cv Jinyou #1)**

Source	DF	SS	MS	F	P
r	2	510	255		
salt	1	1217865	1217865	670.09	0.0015
Error r*salt	2	3635	1817		
Treat	3	21132	7044	7.59	0.0042
salt*Treat	3	6869	2290	2.47	0.1123
Error r*salt*Treat	12	11144	929		
Total	23	1261154			

Grand Mean 1017.8
CV(r*salt) 4.19
CV(r*salt*Treat) 2.99

S27a: **Analysis of Variance Table for Catalase (CAT) after 10 days (Cv Jinyou #1)**

Source	DF	SS	MS	F	P
r	2	206	103		
salt	1	137901	137901	109.71	0.0090
Error r*salt	2	2514	1257		
Treat	3	1353	451	0.24	0.8655
salt*Treat	3	1420	473	0.25	0.8573
Error r*salt*Treat	12	22391	1866		
Total	23	165785			

Grand Mean 417.48
CV(r*salt) 8.49
CV(r*salt*Treat) 10.35

S28a: Analysis of Variance Table for Catalase (CAT) after 20 days (Cv Jinyou #1)

Source	DF	SS	MS	F	P
r	2	571	285		
salt	1	326422	326422	256.16	0.0039
Error r*salt	2	2549	1274		
Treat	3	4331	1444	1.17	0.3630
salt*Treat	3	2040	680	0.55	0.6580
Error r*salt*Treat	12	14850	1237		
Total	23	350762			

Grand Mean 451.12
CV(r*salt) 7.91
CV(r*salt*Treat) 7.80

S29a: Analysis of Variance Table for Catalase (CAT) after 30 days (Cv Jinyou #1)

Source	DF	SS	MS	F	P
r	2	3748	1874		
salt	1	497815	497815	2473.86	0.0004
Error r*salt	2	402	201		
Treat	3	5142	1714	2.39	0.1198
salt*Treat	3	775	258	0.36	0.7830
Error r*salt*Treat	12	8607	717		
Total	23	516488			

Grand Mean 472.99
CV(r*salt) 3.00
CV(r*salt*Treat) 5.66

S30: Analysis of Variance Table for Catalase (CAT) after 40 days (Cv Jinyou #1)

Source	DF	SS	MS	F	P
r	2	754	377		
salt	1	671153	671153	319.74	0.0031
Error r*salt	2	4198	2099		
Treat	3	11900	3967	3.73	0.0418
salt*Treat	3	4810	1603	1.51	0.2623
Error r*salt*Treat	12	12747	1062		
Total	23	705562			

Grand Mean 504.71
CV(r*salt) 9.08
CV(r*salt*Treat) 6.46

S31a: Analysis of Variance Table for Malondialdehyde (MDA) after 10 days (Cv jinyou #1)

Source	DF	SS	MS	F	P
r	2	0.00533	0.00267		

salt	1	3.56282	3.56282	182.44	0.0054
Error r*salt	2	0.03906	0.01953		
Treat	3	0.00872	0.00291	0.20	0.8945
salt*Treat	3	0.10148	0.03383	2.32	0.1266
Error r*salt*Treat	12	0.17462	0.01455		
Total	23	3.89204			

Grand Mean 1.4220
CV(r*salt) 9.83
CV(r*salt*Treat) 8.48

S32a: Analysis of Variance Table for Malondialdehyde (MDA)after 20 days (Cv Jinyou #1)

**Source**	**DF**	**SS**	**MS**	**F**	**P**
r	2	0.2563	0.12817		
salt	1	9.0797	9.07966	32.81	0.0292
Error r*salt	2	0.5535	0.27675		
Treat	3	0.2913	0.09711	1.04	0.4082
salt*Treat	3	0.3389	0.11296	1.22	0.3464
Error r*salt*Treat	12	1.1154	0.09295		
Total	23	11.6351			

Grand Mean 1.9394
CV(r*salt) 27.13
CV(r*salt*Treat) 15.72

S33a: Analysis of Variance Table for Malondialdehyde (MDA) after 30 days (Cv Jinyou #1)

**Source**	**DF**	**SS**	**MS**	**F**	**P**
r	2	0.0639	0.0319		
salt	1	20.8425	20.8425	262.72	0.0038
Error r*salt	2	0.1587	0.0793		
Treat	3	0.3188	0.1063	1.93	0.1788
salt*Treat	3	0.1024	0.0341	0.62	0.6155
Error r*salt*Treat	12	0.6610	0.0551		
Total	23	22.1473			

Grand Mean 2.4914
CV(r*salt) 11.31
CV(r*salt*Treat) 9.42

S34a: Analysis of Variance Table for Malondialdehyde (MDA)after 40 days (Cv Jinyou #1)

**Source**	**DF**	**SS**	**MS**	**F**	**P**
r	2	0.1103	0.0551		
salt	1	34.0946	34.0946	1232.25	0.0008
Error r*salt	2	0.0553	0.0277		
Treat	3	0.2410	0.0803	3.54	0.0482
salt*Treat	3	0.0900	0.0300	1.32	0.3128
Error r*salt*Treat	12	0.2722	0.0227		
Total	23	34.8634			

Grand Mean 2.8457
CV(r*salt) 5.85
CV(r*salt*Treat) 5.29

S35a: **Analysis of Variance Table for AMF colonization percentage**

Source	DF	SS	MS	F	P
r	2	1.20	0.60		
s	1	2787.70	2787.70	663.82	0.0015
Error r*s	2	8.40	4.20		
t	1	223.78	223.78	107.73	0.0005
s*t	1	6.28	6.28	3.02	0.09571
Error r*s*t	4	8.31	2.08		
Total	11	3035.67			

Grand Mean 66.060
CV(r*s) 3.10
CV(r*s*t) 2.18

S36a: **Analysis of Variance Table for Nitrogen conc. in shoot (Cv Jinyou #1)**

Source	DF	SS	MS	F	P
r	2	32.09	16.04		
salt	1	1229.79	1229.79	606.01	0.0016
Error r*salt	2	4.06	2.03		
Treat	3	269.48	89.83	7.83	0.0037
salt*Treat	3	57.46	19.15	1.67	0.2260
Error r*salt*Treat	12	137.60	11.47		
Total	23	1730.48			

Grand Mean 43.821
CV(r*salt) 3.25
CV(r*salt*Treat) 7.73

S37a: **Analysis of Variance Table for Nitrogen conc. in root (Cv Jinyou #1)**

Source	DF	SS	MS	F	P
r	2	119.079	59.539		
salt	1	200.967	200.967	4.94	0.1563
Error r*salt	2	81.333	40.667		
Treat	3	110.905	36.968	1.20	0.3531
salt*Treat	3	18.264	6.088	0.20	0.8965
Error r*salt*Treat	12	371.131	30.928		
Total	23	901.679			

Grand Mean 17.350
CV(r*salt) 36.75
CV(r*salt*Treat) 32.05

S38a: **Analysis of Variance Table for phosphorus conc. in shoot (Cv Jinyou #1)**

Source	DF	SS	MS	F	P
r	2	5.75	2.87		
salt	1	1059.03	1059.03	2038.63	0.0005
Error r*salt	2	1.04	0.52		
Treat	3	105.82	35.27	7.57	0.0042
salt*Treat	3	14.08	4.69	1.01	0.4231
Error r*salt*Treat	12	55.88	4.66		
Total	23	1241.59			

Grand Mean 39.027
CV(r*salt) 1.85
CV(r*salt*Treat) 5.53

S39a: Analysis of Variance Table for phosphorus conc. in root (Cv Jinyou #1)

Source	DF	SS	MS	F	P
r	2	14.32	7.16		
salt	1	2297.42	2297.42	169.47	0.0058
Error r*salt	2	27.11	13.56		
Treat	3	733.36	244.45	23.50	0.0000
salt*Treat	3	86.40	28.80	2.77	0.0875
Error r*salt*Treat	12	124.81	10.40		
Total	23	3283.42			

Grand Mean 98.836
CV(r*salt) 3.73
CV(r*salt*Treat) 3.26

S40a: Analysis of Variance Table for potassium conc. in shoot (Cv Jinyou #1)

Source	DF	SS	MS	F	P
r	2	7.61	3.81		
salt	1	4892.92	4892.92	299.01	0.0033
Error r*salt	2	32.73	16.36		
Treat	3	570.01	190.00	42.39	0.0000
salt*Treat	3	57.65	19.22	4.29	0.0284
Error r*salt*Treat	12	53.78	4.48		
Total	23	5614.70			

Grand Mean 73.857
CV(r*salt) 5.48
CV(r*salt*Treat) 2.87

S41a: Analysis of Variance Table for potassium conc. in root (Cv Jinyou #1)

Source	DF	SS	MS	F	P
r	2	10.08	5.04		
salt	1	4436.47	4436.47	908.41	0.0011
Error r*salt	2	9.77	4.88		
Treat	3	268.52	89.51	16.13	0.0002
salt*Treat	3	1833.72	611.24	110.15	0.0000
Error r*salt*Treat	12	66.59	5.55		
Total	23	6625.15			

Grand Mean 107.89
CV(r*salt) 2.05
CV(r*salt*Treat) 2.18

S42a: Analysis of Variance Table for sodium conc.in shoot (Cv Jinyou #1)

Source	DF	SS	MS	F	P
r	2	4.5	2.2		
salt	1	41247.0	41247.0	2666.16	0.0004
Error r*salt	2	30.9	15.5		
Treat	3	6006.3	2002.1	529.96	0.0000
salt*Treat	3	5008.4	1669.5	441.91	0.0000
Error r*salt*Treat	12	45.3	3.8		
Total	23	52342.4			

Grand Mean 62.977
CV(r*salt) 6.25
CV(r*salt*Treat) 3.09

S43a: **Analysis of Variance Table for sodium conc. in root (Cv Jinyou #1)**

Source	DF	SS	MS	F	P
r	2	23.2	11.6		
salt	1	58443.2	58443.2	12007.4	0.0001
Error r*salt	2	9.7	4.9		
Treat	3	2568.0	856.0	34.05	0.0000
salt*Treat	3	1202.3	400.8	15.94	0.0002
Error r*salt*Treat	12	301.7	25.1		
Total	23	62548.0			

Grand Mean 74.043
CV(r*salt) 2.98
CV(r*salt*Treat) 6.77

S44a: **Analysis of Variance Table for K/Na ratio in shoot (Cv Jinyou #1)**

Source	DF	SS	MS	F	P
r	2	0.2989	0.1494		
salt	1	75.5664	75.5664	500.53	0.0020
Error r*salt	2	0.3019	0.1510		
Treat	3	2.8885	0.9628	29.81	0.0000
salt*Treat	3	0.8471	0.2824	8.74	0.0024
Error r*salt*Treat	12	0.3876	0.0323		
Total	23	80.2903			

Grand Mean 2.3968
CV(r*salt) 16.21
CV(r*salt*Treat) 7.50

S45a: **Analysis of Variance Table for K/Na ratio in root (Cv jinyou #1)**

Source	DF	SS	MS	F	P
r	2	0.4103	0.2052		
salt	1	54.0997	54.0997	313.69	0.0032
Error r*salt	2	0.3449	0.1725		
Treat	3	4.0262	1.3421	4.69	0.0217
salt*Treat	3	3.3454	1.1151	3.90	0.0372
Error r*salt*Treat	12	3.4350	0.2863		
Total	23	65.6615			

Grand Mean 2.4931
CV(r*salt) 16.66
CV(r*salt*Treat) 21.46

S1b: **Analysis of Variance Table for Plant length (Cv CCMC)**

Source	DF	SS	MS	F	P
r	2	39.73	19.87		
salt	1	1218.37	1218.37	171.00	0.0058
Error r*salt	2	14.25	7.12		
Treat	3	328.20	109.40	5.60	0.0123
salt*Treat	3	52.01	17.34	0.89	0.4755
Error r*salt*Treat	12	234.54	19.54		
Total	23	1887.10			

Grand Mean 80.959
CV(r*salt) 3.30
CV(r*salt*Treat) 5.46

S2b: **Analysis of Variance Table for Root length (Cv CCMC)**

Source	DF	SS	MS	F	P
r	2	72.63	36.31		
salt	1	1117.25	1117.25	58.32	0.0167
Error r*salt	2	38.32	19.16		
Treat	3	134.96	44.99	1.24	0.3374
salt*Treat	3	13.88	4.63	0.13	0.9418
Error r*salt*Treat	12	434.41	36.20		
Total	23	1811.45			

Grand Mean 44.065
CV(r*salt) 9.93
CV(r*salt*Treat) 13.65

S3b: **Analysis of Variance Table for Shoot fresh weight (Cv CCMC)**

Source	DF	SS	MS	F	P
r	2	191.90	95.95		
salt	1	5089.89	5089.89	563.02	0.0018
Error r*salt	2	18.08	9.04		
Treat	3	743.54	247.85	24.16	0.0000
salt*Treat	3	251.31	83.77	8.17	0.0031
Error r*salt*Treat	12	123.08	10.26		
Total	23	6417.80			

Grand Mean 90.268
CV(r*salt) 3.33
CV(r*salt*Treat) 3.55

S4b: **Analysis of Variance Table for Shoot dry weight (Cv CCMC)**

Source	DF	SS	MS	F	P
r	2	12.225	6.113		
salt	1	311.905	311.905	38.92	0.0247
Error r*salt	2	16.027	8.013		
Treat	3	71.506	23.835	2.73	0.0901
salt*Treat	3	12.655	4.218	0.48	0.6999
Error r*salt*Treat	12	104.683	8.724		
Total	23	529.001			

Grand Mean 15.183
CV(r*salt) 18.64
CV(r*salt*Treat) 19.45

S5b: **Analysis of Variance Table for Root fresh weight (Cv CCMC)**

Source	DF	SS	MS	F	P
r	2	1.035	0.518		
salt	1	255.323	255.323	57.31	0.0170
Error r*salt	2	8.910	4.455		
Treat	3	62.415	20.805	1.53	0.2584
salt*Treat	3	2.819	0.940	0.07	0.9754
Error r*salt*Treat	12	163.674	13.639		

Total	23	494.176

Grand Mean 16.637
CV(r*salt) 12.69
CV(r*salt*Treat) 22.20

S6b: Analysis of Variance Table for Root dry weight (Cv CCMC)

Source	DF	SS	MS	F	P
r	2	0.3039	0.1519		
salt	1	28.1233	28.1233	2995.83	0.0003
Error r*salt	2	0.0188	0.0094		
Treat	3	4.6224	1.5408	13.17	0.0004
salt*Treat	3	1.0256	0.3419	2.92	0.0774
Error r*salt*Treat	12	1.4037	0.1170		
Total	23	35.4978			

Grand Mean 3.0208
CV(r*salt) 3.21
CV(r*salt*Treat) 11.32

S7b: Analysis of Variance Table for chlorophyll a (Cv CCMC)

Source	DF	SS	MS	F	P
r	2	0.1986	0.0993		
salt	1	57.8283	57.8283	987.07	0.0010
Error r*salt	2	0.1172	0.0586		
Treat	3	8.4940	2.8313	22.15	0.0000
salt*Treat	3	6.9749	2.3250	18.19	0.0001
Error r*salt*Treat	12	1.5337	0.1278		
Total	23	75.1467			

Grand Mean 15.902
CV(r*salt) 1.52
CV(r*salt*Treat) 2.25

S8b: Analysis of Variance Table for chlorophyll b (Cv CCMC)

Source	DF	SS	MS	F	P
r	2	1.0343	0.5172		
salt	1	11.0820	11.0820	47.36	0.0205
Error r*salt	2	0.4680	0.2340		
Treat	3	4.2499	1.4166	2.75	0.0889
salt*Treat	3	1.1247	0.3749	0.73	0.5549
Error r*salt*Treat	12	6.1829	0.5152		
Total	23	24.1419			

Grand Mean 4.6205
CV(r*salt) 10.47
CV(r*salt*Treat) 15.54

S9b: Analysis of Variance Table for total chlorophyll (Cv CCMC)

Source	DF	SS	MS	F	P
r	2	0.401	0.200		
salt	1	119.540	119.540	240.93	0.0041
Error r*salt	2	0.992	0.496		
Treat	3	24.358	8.119	14.02	0.0003
salt*Treat	3	13.616	4.539	7.84	0.0037
Error r*salt*Treat	12	6.950	0.579		

Total	23	165.857

Grand Mean 20.523
CV(r*salt) 3.43
CV(r*salt*Treat) 3.71

S10b: Analysis of Variance Table for Root activity (Cv CCMC)

Source	DF	SS	MS	F	P
r	2	15.507	7.753		
salt	1	459.231	459.231	218.77	0.0045
Error r*salt	2	4.198	2.099		
Treat	3	89.757	29.919	10.50	0.0011
salt*Treat	3	28.640	9.547	3.35	0.0556
Error r*salt*Treat	12	34.207	2.851		
Total	23	631.539			

Grand Mean 22.284
CV(r*salt) 6.50
CV(r*salt*Treat) 7.58

S11b: Analysis of Variance Table for Electrolyte leakage after 20 days (Cv CCMC)

Source	DF	SS	MS	F	P
r	2	2.27	1.137		
salt	1	896.83	896.826	5854.75	0.0002
Error r*salt	2	0.31	0.153		
Treat	3	54.53	18.176	10.41	0.0012
salt*Treat	3	73.54	24.513	14.03	0.0003
Error r*salt*Treat	12	20.96	1.747		
Total	23	1048.43			

Grand Mean 19.985
CV(r*salt) 1.96
CV(r*salt*Treat) 6.61

S12b: Analysis of Variance Table for Electrolyte leakage after 40 days (Cv CCMC)

Source	DF	SS	MS	F	P
r	2	0.279	0.140		
salt	1	677.450	677.450	114.81	0.0086
Error r*salt	2	11.801	5.900		
Treat	3	82.624	27.541	23.30	0.0000
salt*Treat	3	35.337	11.779	9.96	0.0014
Error r*salt*Treat	12	14.185	1.182		
Total	23	821.676			

Grand Mean 12.102
CV(r*salt) 20.07
CV(r*salt*Treat) 8.98

S13b: Analysis of Variance Table for Leaf relative water content after 20 days (Cv CCMC)

Source	DF	SS	MS	F	P
r	2	3.75	1.87		
salt	1	1717.01	1717.01	40.51	0.0238
Error r*salt	2	84.76	42.38		

Treat	3	503.26	167.75	13.98	0.0003
salt*Treat	3	159.64	53.21	4.43	0.0257
Error r*salt*Treat	12	144.00	12.00		
Total	23	2612.42			

Grand Mean 74.512
CV(r*salt) 8.74
CV(r*salt*Treat) 4.65

<u>S14b:</u> Analysis of Variance Table for Leaf relative water content after 40 days (Cv CCMC)

**Source**	**DF**	**SS**	**MS**	**F**	**P**
r	2	5.22	2.61		
salt	1	3155.81	3155.81	4392.73	0.0002
Error r*salt	2	1.44	0.72		
Treat	3	269.72	89.91	14.43	0.0003
salt*Treat	3	87.35	29.12	4.67	0.0219
Error r*salt*Treat	12	74.79	6.23		
Total	23	3594.33			

Grand Mean 53.524
CV(r*salt) 1.58
CV(r*salt*Treat) 4.66

<u>S15b:</u> Analysis of Variance Table for photosynthesis (Cv CCMC)

**Source**	**DF**	**SS**	**MS**	**F**	**P**
r	2	1.2800	0.6400		
salt	1	31.6251	31.6251	82.54	0.0119
Error r*salt	2	0.7663	0.3832		
Treat	3	5.2515	1.7505	6.52	0.0073
salt*Treat	3	2.5041	0.8347	3.11	0.0667
Error r*salt*Treat	12	3.2197	0.2683		
Total	23	44.6469			

Grand Mean 7.5587
CV(r*salt) 8.19
CV(r*salt*Treat) 6.85

<u>S16b:</u> Analysis of Variance Table for stomatal conductance (Cv CCMC)

**Source**	**DF**	**SS**	**MS**	**F**	**P**
r	2	0.00119	0.00060		
salt	1	0.04646	0.04646	48.55	0.0200
Error r*salt	2	0.00191	0.00096		
Treat	3	0.00177	0.00059	1.28	0.3255
salt*Treat	3	0.00282	0.00094	2.04	0.1615
Error r*salt*Treat	12	0.00553	0.00046		

Total	23	0.05969			

Grand Mean 0.2068
CV(r*salt) 14.96
CV(r*salt*Treat) 10.38

S17b: Analysis of Variance Table for intercellular Carbon dioxide conc. (Cv CCMC)

Source	DF	SS	MS	F	P
r	2	13.08	6.54		
salt	1	4999.71	4999.71	731.98	0.0014
Error r*salt	2	13.66	6.83		
Treat	3	1159.11	386.37	40.31	0.0000
salt*Treat	3	171.42	57.14	5.96	0.0100
Error r*salt*Treat	12	115.02	9.59		
Total	23	6472.00			

Grand Mean 381.10
CV(r*salt) 0.69
CV(r*salt*Treat) 1.81

S18b: Analysis of Variance Table for transpiration rate (Cv CCMC)

Source	DF	SS	MS	F	P
r	2	0.0006	0.0003		
salt	1	10.2133	10.2133	9014.75	0.0001
Error r*salt	2	0.0023	0.0011		
Treat	3	0.4203	0.1401	83.93	0.0000
salt*Treat	3	0.1171	0.0390	23.38	0.0000
Error r*salt*Treat	12	0.0200	0.0017		
Total	23	10.7736			

Grand Mean 1.4318
CV(r*salt) 2.35
CV(r*salt*Treat) 2.85

S19b: Analysis of Variance Table for Superoxide dismutase (SOD) after 10 days (Cv CCMC)

Source	DF	SS	MS	F	P
r	2	4461	2231		
salt	1	166236	166236	912.38	0.0011
Error r*salt	2	364	182		
Treat	3	3078	1026	1.02	0.4198
salt*Treat	3	3409	1136	1.12	0.3778
Error r*salt*Treat	12	12122	1010		
Total	23	189670			

Grand Mean 432.81
CV(r*salt) 3.12
CV(r*salt*Treat) 7.34

S20b: Analysis of Variance Table for Superoxide dismutase (SOD) after 20 days (CV CCMC)

Source	DF	SS	MS	F	P
r	2	5824	2912		
salt	1	407003	407003	2472.76	0.0004
Error r*salt	2	329	165		

Treat	3	5180	1727	2.16	0.1462
salt*Treat	3	4063	1354	1.69	0.2216
Error r*salt*Treat	12	9606	801		
Total	23	432006			

Grand Mean 490.44
CV(r*salt) 2.62
CV(r*salt*Treat) 5.77

S21b: Analysis of Variance Table for Superoxide dismutase (SOD) after 30 days (Cv CCMC)

Source	DF	SS	MS	F	P
r	2	4148	2074		
salt	1	366930	366930	776.51	0.0013
Error r*salt	2	945	473		
Treat	3	7237	2412	9.62	0.0016
salt*Treat	3	8502	2834	11.30	0.0008
Error r*salt*Treat	12	3009	251		
Total	23	390772			

Grand Mean 497.06
CV(r*salt) 4.37
CV(r*salt*Treat) 3.19

S22b: Analysis of Variance Table for Superoxide dismutase (SOD) after 40 days (Cv CCMC)

Source	DF	SS	MS	F	P
r	2	1416	708		
salt	1	160586	160586	116.09	0.0085
Error r*salt	2	2767	1383		
Treat	3	9920	3307	12.36	0.0006
salt*Treat	3	1246	415	1.55	0.2521
Error r*salt*Treat	12	3211	268		
Total	23	179145			

Grand Mean 488.54
CV(r*salt) 7.61
CV(r*salt*Treat) 3.35

S23b: Analysis of Variance Table for Peroxidase (POD) after 10 days (Cv CCMC)

Source	DF	SS	MS	F	P
r	2	7135	3568		
salt	1	1030999	1030999	448.86	0.0022
Error r*salt	2	4594	2297		
Treat	3	10627	3542	2.59	0.1014
salt*Treat	3	5199	1733	1.27	0.3299
Error r*salt*Treat	12	16420	1368		
Total	23	1074975			

Grand Mean 1000.2
CV(r*salt) 4.79
CV(r*salt*Treat) 3.70

S24b: Analysis of Variance Table for Peroxidase (POD) after 20 days (Cv CCMC)

Source	DF	SS	MS	F	P
r	2	2776	1388		
salt	1	923067	923067	4062.58	0.0002
Error r*salt	2	454	227		
Treat	3	8129	2710	2.26	0.1343
salt*Treat	3	6580	2193	1.83	0.1961
Error r*salt*Treat	12	14416	1201		
Total	23	955422			

Grand Mean 1029.6
CV(r*salt) 1.46
CV(r*salt*Treat) 3.37

S25b: Analysis of Variance Table for Peroxidase (POD) after 30 days (Cv CCMC)

Source	DF	SS	MS	F	P
r	2	1558	779		
salt	1	1008143	1008143	1632.13	0.0006
Error r*salt	2	1235	618		
Treat	3	6056	2019	1.88	0.1863
salt*Treat	3	2305	768	0.72	0.5607
Error r*salt*Treat	12	12865	1072		
Total	23	1032163			

Grand Mean 1042.5
CV(r*salt) 2.38
CV(r*salt*Treat) 3.14

S26b: Analysis of Variance Table for Peroxidase (POD) after 40 days (Cv CCMC)

Source	DF	SS	MS	F	P
r	2	38	19		
salt	1	857529	857529	357.65	0.0028
Error r*salt	2	4795	2398		
Treat	3	15973	5324	8.38	0.0028
salt*Treat	3	8096	2699	4.25	0.0291
Error r*salt*Treat	12	7623	635		
Total	23	894055			

Grand Mean 1044.1
CV(r*salt) 4.69
CV(r*salt*Treat) 2.41

S27b: Analysis of Variance Table for Catalase (CAT) after 10 days (Cv CCMC)

Source	DF	SS	MS	F	P
r	2	1236	618		
salt	1	122132	122132	203.38	0.0049
Error r*salt	2	1201	601		
Treat	3	5412	1804	0.62	0.6177
salt*Treat	3	400	133	0.05	0.9865
Error r*salt*Treat	12	35146	2929		
Total	23	165527			

Grand Mean 418.79
CV(r*salt) 5.85
CV(r*salt*Treat) 12.92

S28b: **Analysis of Variance Table for Catalase (CAT) after 20 days (Cv CCMC)**

Source	DF	SS	MS	F	P
r	2	1481	740		
salt	1	301502	301502	6214.05	0.0002
Error r*salt	2	97	49		
Treat	3	3391	1130	1.03	0.4130
salt*Treat	3	3392	1131	1.03	0.4129
Error r*salt*Treat	12	13137	1095		
Total	23	323000			

Grand Mean 473.75
CV(r*salt) 1.47
CV(r*salt*Treat) 6.98

S29b: **Analysis of Variance Table for Catalase (CAT) after 30 days (Cv CCMC)**

Source	DF	SS	MS	F	P
r	2	2550	1275		
salt	1	529672	529672	856126	0.0000
Error r*salt	2	1	1		
Treat	3	15528	5176	6.39	0.0078
salt*Treat	3	3198	1066	1.32	0.3149
Error r*salt*Treat	12	9724	810		
Total	23	560673			

Grand Mean 518.45
CV(r*salt) 0.15
CV(r*salt*Treat) 5.49

S30b: **Analysis of Variance Table for Catalase (CAT) after 40 days (Cv CCMC)**

Source	DF	SS	MS	F	P
r	2	2418	1209		
salt	1	807135	807135	337.18	0.0030
Error r*salt	2	4788	2394		
Treat	3	13235	4412	8.21	0.0031
salt*Treat	3	2367	789	1.47	0.2727
Error r*salt*Treat	12	6450	537		
Total	23	836393			

Grand Mean 558.85
CV(r*salt) 8.75
CV(r*salt*Treat) 4.15

S31b: **Analysis of Variance Table for Malondialdehyde (MDA) after 10 days (Cv CCMC)**

Source	DF	SS	MS	F	P
r	2	0.00079	0.00039		
salt	1	1.77858	1.77858	6221.53	0.0002
Error r*salt	2	0.00057	0.00029		
Treat	3	0.02119	0.00706	1.39	0.2920
salt*Treat	3	0.02145	0.00715	1.41	0.2874
Error r*salt*Treat	12	0.06077	0.00506		
Total	23	1.88336			

Grand Mean 1.3892
CV(r*salt) 1.22
CV(r*salt*Treat) 5.12

S32b: Analysis of Variance Table for Malondialdehyde (MDA) after 20 days (Cv CCMC)

Source	DF	SS	MS	F	P
r	2	0.03334	0.01667		
salt	1	8.97962	8.97962	366.27	0.0027
Error r*salt	2	0.04903	0.02452		
Treat	3	0.16710	0.05570	2.16	0.1455
salt*Treat	3	0.17362	0.05787	2.25	0.1353
Error r*salt*Treat	12	0.30909	0.02576		
Total	23	9.71180			

Grand Mean 2.0506
CV(r*salt) 7.64
CV(r*salt*Treat) 7.83

S33b: Analysis of Variance Table for Malondialdehyde (MDA) after 30 days (Cv CCMC)

Source	DF	SS	MS	F	P
r	2	0.0908	0.0454		
salt	1	15.9753	15.9753	456.66	0.0022
Error r*salt	2	0.0700	0.0350		
Treat	3	0.2734	0.0911	3.32	0.0568
salt*Treat	3	0.1734	0.0578	2.11	0.1529
Error r*salt*Treat	12	0.3294	0.0275		
Total	23	16.9123			

Grand Mean 2.4549
CV(r*salt) 7.62
CV(r*salt*Treat) 6.75

S34b: Analysis of Variance Table for Malondialdehyde (MDA) after 40 days (Cv CCMC)

Source	DF	SS	MS	F	P
r	2	0.0819	0.0409		
salt	1	27.3330	27.3330	2744.41	0.0004
Error r*salt	2	0.0199	0.0100		
Treat	3	0.1617	0.0539	1.36	0.3019
salt*Treat	3	0.0854	0.0285	0.72	0.5598
Error r*salt*Treat	12	0.4755	0.0396		
Total	23	28.1575			

Grand Mean 2.8573
CV(r*salt) 3.49
CV(r*salt*Treat) 6.97

S35b: Analysis of Variance Table for AMF colonization percentage

Source	DF	SS	MS	F	P
r	2	7.94	3.97		
s	1	1330.99	1330.99	167.84	0.0059
Error r*s	2	15.86	7.93		
t	1	76.00	76.00	41.11	0.0030

s*t	1	0.51	0.51	0.28	0.2264
Error r*s*t	4	7.40	1.85		
Total	11	1438.71			

Grand Mean 65.565
CV(r*s) 4.30
CV(r*s*t) 2.07

S36b: Analysis of Variance Table for Nitrogen conc. in shoot (Cv CCMC)

Source	DF	SS	MS	F	P
r	2	0.37	0.184		
salt	1	521.64	521.644	24.84	0.0380
Error r*salt	2	42.00	21.001		
Treat	3	298.34	99.446	5.09	0.0167
salt*Treat	3	36.13	12.043	0.62	0.6172
Error r*salt*Treat	12	234.24	19.520		
Total	23	1132.72			

Grand Mean 42.694
CV(r*salt) 10.73
CV(r*salt*Treat) 10.35

S37b: Analysis of Variance Table for Nitrogen Conc. in root (Cv CCMC)

Source	DF	SS	MS	F	P
r	2	1.105	0.5525		
salt	1	97.020	97.0202	3.95	0.1853
Error r*salt	2	49.157	24.5787		
Treat	3	32.943	10.9810	1.78	0.2050
salt*Treat	3	0.742	0.2474	0.04	0.9888
Error r*salt*Treat	12	74.156	6.1797		
Total	23	255.124			

Grand Mean 9.8638
CV(r*salt) 50.26
CV(r*salt*Treat) 25.20

S38b: Analysis of Variance Table for Phosphorus conc. in shoot (Cv CCMC)

Source	DF	SS	MS	F	P
r	2	2.59	1.29		
salt	1	1034.61	1034.61	533.13	0.0019
Error r*salt	2	3.88	1.94		
Treat	3	190.09	63.36	18.62	0.0001
salt*Treat	3	18.02	6.01	1.77	0.2072
Error r*salt*Treat	12	40.83	3.40		
Total	23	1290.01			

Grand Mean 44.203
CV(r*salt) 3.15
CV(r*salt*Treat) 4.17

S39b: **Analysis of Variance Table for phosphorus Conc. in root (Cv CCMC)**

Source	DF	SS	MS	F	P
r	2	59.76	29.88		
salt	1	6187.73	6187.73	629.22	0.0016
Error r*salt	2	19.67	9.83		
Treat	3	1522.77	507.59	11.16	0.0009
salt*Treat	3	157.76	52.59	1.16	0.3667
Error r*salt*Treat	12	545.95	45.50		
Total	23	8493.63			

Grand Mean 114.91
CV(r*salt) 2.73
CV(r*salt*Treat) 5.87

S40b: **Analysis of Variance Table for Potassium Conc. in shoot (Cv CCMC)**

Source	DF	SS	MS	F	P
r	2	7.53	3.76		
salt	1	4041.44	4041.44	1280.37	0.0008
Error r*salt	2	6.31	3.16		
Treat	3	551.68	183.89	86.10	0.0000
salt*Treat	3	83.15	27.72	12.98	0.0004
Error r*salt*Treat	12	25.63	2.14		
Total	23	4715.74			

Grand Mean 77.385
CV(r*salt) 2.30
CV(r*salt*Treat) 1.89

S41b: **Analysis of Variance Table for potassium Conc. in root (Cv CCMC)**

Source	DF	SS	MS	F	P
r	2	54.08	27.04		
salt	1	5530.99	5530.99	2398.06	0.0004
Error r*salt	2	4.61	2.31		
Treat	3	94.47	31.49	2.02	0.1654
salt*Treat	3	928.42	309.47	19.82	0.0001
Error r*salt*Treat	12	187.36	15.61		
Total	23	6799.94			

Grand Mean 103.67
CV(r*salt) 1.46
CV(r*salt*Treat) 3.81

S42b: **Analysis of Variance Table for sodium conc. in shoot (Cv CCMC)**

Source	DF	SS	MS	F	P
r	2	17.1	8.5		
salt	1	36075.4	36075.4	585.50	0.0017
Error r*salt	2	123.2	61.6		
Treat	3	3174.4	1058.1	83.51	0.0000
salt*Treat	3	2220.1	740.0	58.40	0.0000
Error r*salt*Treat	12	152.1	12.7		
Total	23	41762.3			

Grand Mean 61.424
CV(r*salt) 12.78
CV(r*salt*Treat) 5.80

S43b: **Analysis of Variance Table for sodium Conc. in root (Cv CCMC)**

Source	DF	SS	MS	F	P
r	2	8.5	4.2		
salt	1	58428.1	58428.1	6064.07	0.0002
Error r*salt	2	19.3	9.6		
Treat	3	1839.0	613.0	105.42	0.0000
salt*Treat	3	913.1	304.4	52.34	0.0000
Error r*salt*Treat	12	69.8	5.8		
Total	23	61277.7			

Grand Mean 73.204
CV(r*salt) 4.24
CV(r*salt*Treat) 3.29

S44b: **Analysis of Variance Table for K/Na ratio in shoot (CV CCMC)**

Source	DF	SS	MS	F	P
r	2	0.1770	0.0885		
salt	1	73.8514	73.8514	473.43	0.0021
Error r*salt	2	0.3120	0.1560		
Treat	3	4.2195	1.4065	7.56	0.0042
salt*Treat	3	1.7941	0.5980	3.21	0.0616
Error r*salt*Treat	12	2.2331	0.1861		
Total	23	82.5871			

Grand Mean 2.4327
CV(r*salt) 16.24
CV(r*salt*Treat) 17.73

S45b: **Analysis of Variance Table for K/Na ratio in root (Cv CCMC)**

Source	DF	SS	MS	F	P
r	2	0.3317	0.1659		
salt	1	47.7795	47.7795	360.85	0.0028
Error r*salt	2	0.2648	0.1324		
Treat	3	2.5758	0.8586	31.33	0.0000
salt*Treat	3	1.9665	0.6555	23.92	0.0000
Error r*salt*Treat	12	0.3289	0.0274		
Total	23	53.2474			

Grand Mean 2.3873
CV(r*salt) 15.24
CV(r*salt*Treat) 6.93
